# Morin incorporated polysaccharide–protein (psyllium–keratin) hydrogel scaffolds accelerate diabetic wound healing in Wistar rats†

**DOI:** 10.1039/c7ra10334d

**Published:** 2018-01-09

**Authors:** Thangavel Ponrasu, Praveen Krishna Veerasubramanian, Ramya Kannan, Selvakumar Gopika, Lonchin Suguna, Vignesh Muthuvijayan

**Affiliations:** Department of Biotechnology, Bhupat and Jyoti Mehta School of Biosciences, Indian Institute of Technology Madras Chennai 600036 India vigneshm@iitm.ac.in +91-44-2257-4102 +91-44-2257-4123; Department of Chemistry, Indian Institute of Technology Madras Chennai 600036 India; Department of Biochemistry, CSIR-Central Leather Research Institute, Council of Scientific and Industrial Research Adyar Chennai 600020 India

## Abstract

Chronic wounds cost several billion dollars of public healthcare spending annually and continue to be a persistent threat globally. Several treatment methods have been explored, and all of them involve covering up the wound with therapeutic dressings that reduce inflammation and accelerate the healing process. In this present study, morin (MOR) was loaded onto hydrogel scaffolds prepared from psyllium seed husk polysaccharide (PSH), and human hair keratins (KER) crosslinked with sodium trimetaphosphate. ATR-FTIR confirmed the presence of the constituent chemical ingredients. SEM images of the scaffold surface reveal a highly porous architecture, with about 80% porosity measured by liquid displacement measurement, irrespective of the morin concentration. Swelling assays carried out on the scaffolds portray an ability to absorb up to seven times their dry weight of fluids. This makes them attractive for guiding moist wound healing on medium exuding wounds. An Alamar blue assay of NIH/3T3 fibroblast cells shows that cell viability decreases in the first 24 h but recovers to 85% in comparison to a control after 48 h. SEM images of fibroblast cells grown on the scaffolds confirm cellular attachment. An *in vivo* diabetic wound healing study showed that PSH + KER + MOR scaffold treatment significantly reduced the re-epithelialization time (*p* < 0.01) and enhanced the rate of wound contraction (*p* < 0.001), by accelerating collagen synthesis in diabetic rats compared to controls.

## Abstract 2

Morin loaded polysaccharide–protein composite scaffolds enhance diabetic wound healing.

## Graphical abstract

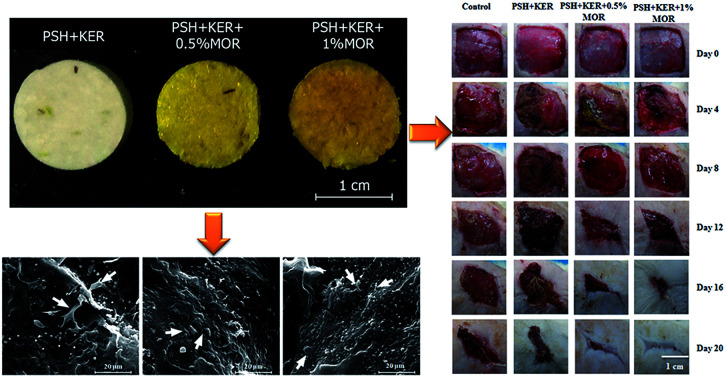

## Introduction

Chronic wounds are those dermal wounds where inflammation continues unabated for more than 14 days.^1^ The quick progress of the phases of initial hemostasis, inflammation, proliferation, and remodeling chronicles an unremarkable healing process. Chronic wounds and associated hospitalization encumber public healthcare with expenses running into several billions of dollars annually.^2^ The issue assumes significance in light of mounting lifestyle diseases including diabetes mellitus and obesity.

Hydrogels are the class of materials capable of absorption of fluid, several times their weight on a dry basis while exhibiting structural integrity and non-dissolution. Hydrogels are capable of maintaining a moist environment when applied on wounds, thus paving the way for an accelerated healing process.^3^ Their tendency towards moisture uptake makes them attractive for use in medium exuding wounds. Recent literature findings show that many hydrogels are currently being studied for the treatment of chronic wounds. Soluble QHREDGS (glutamine–histidine–arginine–glutamic acid–aspartic acid–glycine–serine) peptide modified hydrogels,^4^ dextran based hydrogels,^5^ superoxide dismutase releasing composite hydrogel of chitosan/heparin/poly(γ-glutamic acid),^6^ sustained release of stromal cell derived factor-1 from thermoresponsive hydrogel,^7^ keratin biomaterial hydrogel,^8^ and l-glutamic acid loaded hydrogels^9^ have all shown promising results.

Human hair derived proteins have been found an attractive biomaterial for tissue engineering applications. Keratin is one of the toughest, natural biomaterial, comparable to chitin. Proteins are often chosen as wound dressings for enabling positive cell–cell, cell–extracellular matrix, and cell–material interactions. Keratins derived from human hair (KER) have been fabricated into hydrogels for neural tissue engineering as neuronal conduits. They have been demonstrated to actuate axonal regeneration and functional restoration of peripheral nerve injuries of murine tibial and leporine sciatic nerves.^10–12^ Hydrogels from KER have been validated in a mortal hepatic wound model in rabbits, where they showed excellent utility as a hemostatic biomaterial.^13^ Keratin derived from wool, and its derivatives, have been fabricated as hydrogels and studied for application in controlled drug release. The study demonstrated the sustained release of drugs loaded *in vitro* over a period of 72 h.^14^ A study of KER hydrogels has shown them to support fibroblast proliferation, in a performance comparable to that of collagen hydrogels. This hints at the suitability of KER hydrogels for soft tissue regeneration applications.^15^

Polysaccharides are often used in wound dressings for imparting bulk and mechanical strength. Common examples include chitosan,^16^ cellulose,^17^ starch,^18^ glucomannan,^19^ alginates,^20^ and pullulan.^21^ Psyllium seed husk (PSH) is a complex polysaccharide that is obtained from the herbaceous *Plantago ovate*. The seed husk yields hydrophilic mucilage that finds use as a food thickener, anti-diarrheal agent, and therapy for Crohn's disease and irritable bowel syndrome owing to remarkable gelling potential. The complex arabinoxylan in PSH comprises of highly branched and densely substituted β-(1→4) linked xylopyranose (50–54%) backbones, attached with single arabinofuranose (17–20%) or xylopyranose, or short chains of these at positions 2 and 3. There also exist additional residues, such as rhamnose (3–5%), and galacturonic acid (5–8%) in these side chains.^22^ PSH had been incorporated earlier in wound dressings and studied, in embodiments blended with alginate/silver,^23^ and silk fibroin.^24^ These wound dressings have been demonstrative of the biocompatible and beneficial nature of PSH as a biomaterial.

Morin (MOR), chemically 3,5,7,2′,4′-pentahydroxyflavone, is a plant-derived flavonol notable for antioxidant activity against lipid peroxidation.^25,26^ Morin has also been found to protect rat hepatocytes against hyperglycemia-induced apoptosis, by decreasing reactive oxygen species levels and oxidative stress associated damage.^27^ Morin is also a recognized anti-inflammatory agent, acting by inhibiting cyclooxygenase and lipoxygenase isoforms instrumental in inflammatory processes.^28^ Morin may also be capable of protective effects against cardiovascular diseases owing to curbing of oxidation of low-density lipoproteins by copper ions and macrophages *in vitro*, thereby preventing atherogenesis.^28^ Morin has been reported to prevent the deleterious effects of hyperammonemia in rats.^29^ Morin has also been found to restrict amyloid formation, and could potentially be suitable for treatment of type 2 diabetes and Alzheimer's disease.^30^

Psyllium, the base carbohydrate polymer, acts as the gelling agent that can absorb wound exudates. Keratin is a natural protein that can improve vascularization and collagen synthesis. Morin can protect the wound site through its antioxidant and antibacterial properties to avoid the contamination in chronic wounds. As these components provide important properties required for enhancing wound healing, we hypothesize that a scaffold prepared with PSH, KER, and MOR will provide a synergistic effect on enhancing diabetic wound healing. Therefore, the current work aims to evaluate the topical effects of morin on diabetic cutaneous wounds and healing. KER–PSH blend hydrogel scaffolds, loaded with morin (MOR) of varying concentrations have been fabricated by freeze-drying technique. The chemically crosslinked scaffolds have been explored in this study for their ability to heal diabetic wounds. Sodium trimetaphosphate, a food safe crosslinker that works by forging phosphate linkages between intermolecular hydroxyl groups, was the chemical crosslinker used in this study.^31^

## Materials and methods

### Materials

Psyllium was purchased from the Sat-Isabgol Factory, India. NIH/3T3 fibroblast cells were procured from the National Centre for Cell Science, India. Morin and sodium trimetaphosphate (STMP) were purchased from Sigma-Aldrich. ATCC25922 (*Escherichia coli*) and ATCC25923 (*Staphylococcus aureus*) reference strains were employed for antibacterial assays. Male Wistar albino rats were purchased from King Institute of Preventive Medicine and Research, India.

### Human hair protein extraction

Human hair obtained from a local salon was cleaned, and processed for protein extraction by sodium sulfide reduction method.^32^ Hair was delipidized with chloroform–methanol (2 : 1) mixture. 5 g of chopped hair, mixed with 100 ml of 125 mM sodium sulfide solution, was incubated for 4 h at 40 °C. The filtrate was dialyzed with distilled water over a period of 3–4 days. The dialyzed material was lyophilized to obtain the human hair proteins. The proteins were analyzed with an SDS-PAGE, the results of which showed abundant proteins corresponding to alpha-keratins visualized as two prominent bands in the range of 40–60 kDa, as described previously.^32^ The matrix proteins of lower molecular weights (15–30 kDa) were not visualized. The human hair proteins, predominantly keratins, are henceforth referred as KER.

### Scaffold fabrication

Hydrogel scaffolds were formulated incorporating varying concentrations of morin. 400 mg each of KER and PSH, and varying weights of morin (0, 100, and 200 mg) were dissolved in 18 ml of distilled water. 1 ml each of 15% STMP and 30% NaOH were added to the admixture which was then poured into Teflon molds, kept at −20 °C for 12 h, and lyophilized (Christ Alpha 1-2 LD Freeze Dryer, UK). The freeze-dried product was washed thoroughly with distilled water to remove the NaOH, and subsequently re-lyophilized to obtain porous hydrogel scaffolds. The scaffolds obtained have been termed as PSH + KER, PSH + KER + 0.5% MOR, and PSH + KER + 1% MOR, based on the morin concentration of the preparatory solution.

### Physicochemical characterization

#### ATR-IR spectroscopy and scanning electron microscopy

The identity of various chemical entities in the scaffolds was confirmed by using ATR-FTIR (Bruker Alpha) over the 4000–500 cm^−1^ range. Surface topology and cross-sectional features of gold-sputtered scaffolds were observed using SEM (FEI Quanta 200).

#### Swelling

The capacity of the hydrogel scaffolds for fluid uptake was measured by immersion in phosphate buffered saline (PBS).^33^ Scaffolds sections were weighed (*W*_d_) and placed in PBS (pH 7.4) at 37 °C. The degree of swelling was measured at different time points. The surface was wiped gently with blotting paper to eliminate surface-adsorbed fluid, and the scaffolds weighed again (*W*_w_). The degree of swelling defines the capacity for fluid uptake.
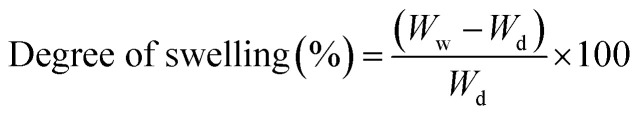


#### Porosity

The porosity of the scaffolds was measured using a simple liquid displacement technique, where scaffold sections of defined volume (*V*) were placed in ethanol for one hour in vacuum.^34,35^ This allows for the saturation of pores. Porosity of the scaffold sections weighed before (*W*_i_), and after (*W*_f_) immersion in alcohol is presented by the following formula,
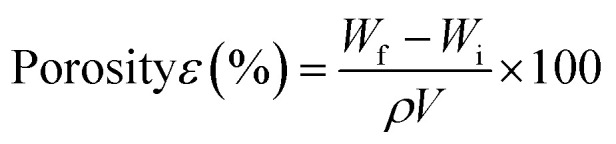
where *ρ* is the density of ethanol.

#### Antioxidant activity

Antioxidant capability of the hydrogel scaffolds was demonstrated using 2,2′-diphenyl-1-picrylhydrazyl (DPPH) free radical scavenging assay under buffered condition.^36,37^ 6 mm diameter discs of the scaffolds, each weighing about 2 mg, were placed in 1 ml of 175 μM DPPH in methanol, buffered to pH 5.5. After 20 min of incubation in dark, the optical density of the solution was quantified at 517 nm (*A*_Sample_), keeping buffered DPPH solution as the control solution (*A*_DPPH_). The ability of the scaffold to bleach the DPPH solution is interpreted as its potential to scavenge free radicals.
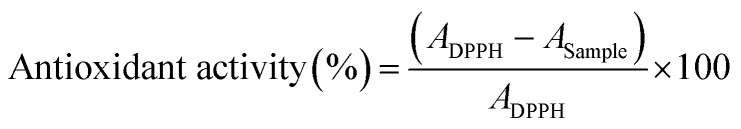


#### Antibacterial activity

Antibacterial activity of the hydrogel scaffolds was assessed by incubating the bacterial culture in presence of the scaffolds, followed by colony count.^34^ In brief, *E. coli* and *S. aureus* bacteria were incubated in sterile lysogeny broth until the reach of log phase. The scaffolds were cut into rectangular sections weighing 50 mg and added to 10 ml of the culture. Post incubation for 3 h at 37 °C, the culture was serially diluted and plated on Luria agar. Viable colonies formed after 24 h were counted to provide the colony forming units (CFU ml^−1^).

#### Cell culture studies

NIH/3T3 murine fibroblast cells were used for *in vitro* biological evaluation studies. Cells were cultured in DMEM with 10% FBS and 1% penicillin–streptomycin, incubated in a humidified, 5% CO_2_ atmosphere at 37 °C. Subconfluent cells (about 80% confluency) were harvested post trypsin treatment, enumerated, and employed for the subsequent assays of cell viability and attachment.

#### Cell viability

Cell viability was quantified using Alamar blue assay.^38^ Briefly, scaffolds discs of 15 mm diameter were UV sterilized overnight, placed in 24-well plates, and immersed in DMEM with 10% FBS-containing medium for 24 h. The medium was then removed, and the scaffolds were washed with PBS. Cells were seeded at a density of 10^4^ cells per well and provided with fresh medium before incubation for 24 and 48 h. Subsequently, 10 μl of Alamar blue stock solution (5 mg ml^−1^) was added per 500 μl of media to each well and incubated at 37 °C, 5% CO_2_ for 4 h. 100 μl of the solution was transferred from each well to a fresh 96-well plate. The optical density was read using a microplate reader at wavelengths of 570 nm and 595 nm. Controls with just media and dye were also incubated for the same period, and measured. Cell viability was estimated based on the degree of reduction in Alamar blue.

#### Cell attachment

SEM imaging was performed on fibroblast cells incubated in the presence of scaffolds to visualize the attachment and proliferation of cells on them. Fibroblast cells were maintained for 48 h in the presence of scaffolds, in the manner described above for cell viability assay. Briefly, the NIH 3T3 cells were seeded on the porous scaffolds and cultured for 48 h in a 24-well plate at a concentration of 1 × 10^4^ cells per well. After 48 h of incubation, the scaffolds were rinsed with PBS solution and fixed with 2.5% glutaraldehyde for 1 h. Then, the samples were thoroughly washed with PBS and sequentially dehydrated in a graded-ethanol series (50%, 70%, 95% and 100%) and lyophilized. The cell attachment in scaffolds was examined using scanning electron microscopy (SEM) and images have been taken.^39^

### *In vivo* studies

#### Animal grouping and maintenance

Healthy male Wistar albino rats with body mass greater than 200 g were selected for demonstration of diabetic wound healing *in vivo*. The animals were lodged in wire topped cages with sterile rice hull bedding. Rats were allowed access to clean feed and water *ad libitum* and placed under controlled 12/12 light cycle at 24–26 °C. Formal approval from the Institutional Animal Ethics Committee (IAEC) was secured (IAEC-01/2016(b)/02.12.2016), and the experiments carried out as per the guidelines laid down by the Committee for the Purpose of Control And Supervision of Experiments on Animals (CPCSEA), Ministry of Environment, Government of India. Rats were segregated into four groups, each comprising of five animals:

Group I – control animals, dressed with non-medicated cotton gauze; group II – rats dressed with PSH + KER scaffolds; group III – rats dressed with PSH + KER + 0.50% MOR scaffolds; group IV – rats dressed with PSH + KER + 1% MOR scaffolds.

#### Diabetic excision wound model

Diabetes was induced in rats by administration of streptozotocin, a drug that causes the destruction of pancreatic β-cells.^40^ In fasting rats, intraperitoneal administration of nicotinamide (110 mg kg^−1^ body weight) was followed by a single intraperitoneal shot of streptozotocin (50 mg kg^−1^ body weight) in cold 0.1 M citrate buffer (pH 4.5), after a 15-minute interval. The tail vein blood glucose levels were monitored periodically using a digital glucometer to ascertain sustained hyperglycemia. Wound creation was carried out 2 weeks after diabetes induction. Rats were anesthetized by a single intraperitoneal injection of sodium thiopentane (50 mg kg^−1^ body weight). A 2 cm × 2 cm full thickness open excision wound was made on the back of the rat as reported in our earlier experiments.^41^ The control rats were treated with a normal gauze dressing and the other groups of rats treated with PSH + KER, PSH + KER + 0.50% MOR, and PSH + KER + 1% MOR scaffold dressings. These dressings were replaced every four days until the wounds healed completely. Wound dressings were removed for taking the digital images of the healed wounds. Granulation tissues were collected on days 4, 8 and 12 for further studies. The rate of wound contraction was traced and photographed at regular intervals till completion. Granulation tissues formed on days 4, 8 were removed and used for the collagen estimation. Animals were euthanized by CO_2_ asphyxiation on the conclusion of experimentation. The period of epithelialization is defined as the time period for complete closure of the wounds to occur. The reduction in wound size was determined by planimetrically and is represented as the decrease in wound area compared to the initial wound area.
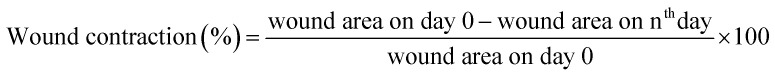
where, *n* is 4, 8, 12, 16, and 20 days.

#### Collagen estimation

An estimation of collagen content in the granulation tissue collected was carried out based on the hydroxyproline index, using a previously described method by Woessner.^42^ Tissue samples were defatted in a 2 : 1 (v/v) chloroform : methanol mixture, and then frozen in acetone. Samples were then weighted, and hydrolyzed in 6 N HCl for 18 h at 110 °C. They were evaporated to dryness and diluted using double distilled water. Hydroxyproline content was measured spectrophotometrically at 557 nm. Collagen content is estimated indirectly as below.Collagen = hydroxyproline × 7.46

#### Histopathology

Granulation tissue samples collected from the animals were fixed in 10% formalin–saline solution, dehydrated with a graded ethanol series, cleared in xylene and embedded in paraffin wax. Samples were sectioned to a thickness of 5 μm and stained with hematoxylin & eosin (H&E) and Masson's trichrome dyes. The stained sections were studied under a light microscope for the observation of histopathological indicators.

#### Statistical analysis

Data obtained from all experiments were analyzed using one-way ANOVA with multiple comparisons using Tukey's test in GraphPad Prism (GraphPad software, CA). All experiments were performed in triplicates. Graphical data is represented as mean ± standard deviation. Significance of data is represented as * (*p* < 0.05), ** (*p* < 0.01), and *** (*p* < 0.001).

## Results and discussion

### Physiochemical characterizations

**Fig. 1:**
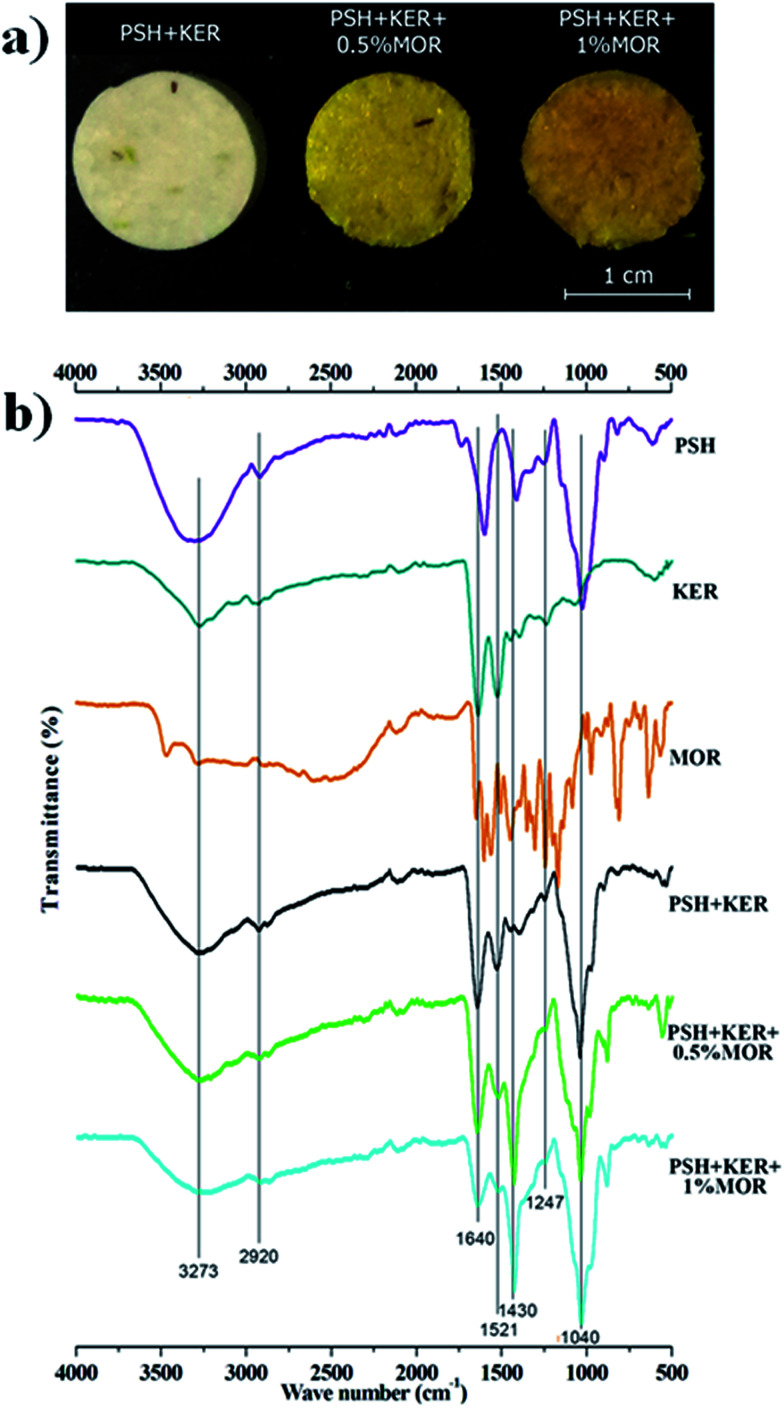
(a) Appearance of the fabricated scaffolds; (b) ATR-FTIR spectra of the materials and fabricated scaffolds.

Visually, the scaffolds incorporated with morin had a yellow appearance (Fig. 1a). Also notable was the increase in the intensity of the color, with an increase of morin concentration. The IR spectra of the scaffolds gave insight on the chemical groups present in the scaffolds, and confirm the presence of the constituting molecules (Fig. 1b). A broad absorption band visible at around 3282 cm^−1^ can be assigned to O–H stretching vibration of hydrogen-bonded hydroxyl groups in PSH. Peaks at around 2920 cm^−1^, 1640 cm^−1^, and 1040 cm^−1^ may be assigned to stretching vibrations of C–H, C=O (of galacturonic acid residues), and C–O–C linkages respectively in PSH.^43^ Characteristic backbone bending of PSH observed at 895 cm^−1^, and 611 cm^−1^.^43^ Human hair keratin produced N–H, and O–H stretching vibrations near 3282 cm^−1^.^32^ C–H stretching vibration of keratin was noticed around 2920 cm^−1^. Bands characteristic in proteins, the amide A (3283–3259 cm^−1^), amide I (1644–1640 cm^−1^), amide II (1527–1519 cm^−1^), and amide III (around 1247 cm^−1^) were all identifiable.^32^ MOR characteristic peaks at 2920 cm^−1^ (C–H stretching), 1640 cm^−1^ (ketone C=O stretching), 1527–1519 cm^−1^ (stretching C=C vibrations of aromatic ring), and 1040–1035 cm^−1^ (C–O–C stretching) were all visualized.^44,45^ Phosphate bridges formed by crosslinking of polymer by STMP is responsible for the characteristic peaks caused by stretching vibrations of P=O, and P–O at 1150–1100 cm^−1^.^43^

IR spectra of the scaffolds confirm the incorporation of the constituents. The presence of characteristic peaks of P=O and P–O stretching at 1150–1100 cm^−1^ is indicative of phosphate bridges formed by the crosslinking agent STMP. There was no appreciable shifting of bands observed in the IR spectra. SEM images of the MOR scaffolds are indicative of its microporous architecture. The porous nature of scaffolds plays an important role in allowing attachment of cells and subsequent proliferation. Porosity measurements corroborate the observations of SEM analysis.

**Fig. 2:**
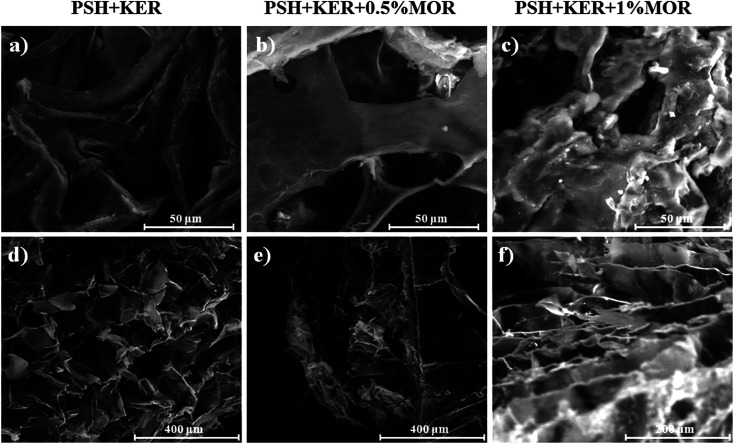
SEM images displaying the surface morphology and microporous nature of (a) PSH + KER, (b) PSH + KER + 0.5% MOR, and (c) PSH + KER + 1% MOR scaffolds; SEM images showing the cross-sectional appearance of (d) PSH + KER, (e) PSH + KER + 0.5% MOR, and (f) PSH + KER + 1% MOR scaffolds.

SEM analysis of the MOR scaffolds shows a highly porous architecture, with a multitude of surface features, and a spongy cross-section that hints at interconnected internal pore networks (Fig. 2). The microporous nature of the scaffolds can elicit the attachment of fibroblast cells, and facilitate its proliferation. Swelling of the scaffolds was assessed by PBS absorption.

We observed that the morin release occurred within the first few hours and it follows a burst pattern (Fig. S2†). *In vitro* biodegradation studies showed that PSH + KER, PSH + KER + 0.5% MOR and PSH + KER + 1% MOR scaffolds were quite stable for 60 days (Fig. S3†). The scaffolds showed minimal degradation during the *in vivo* studies also.

**Fig. 3:**
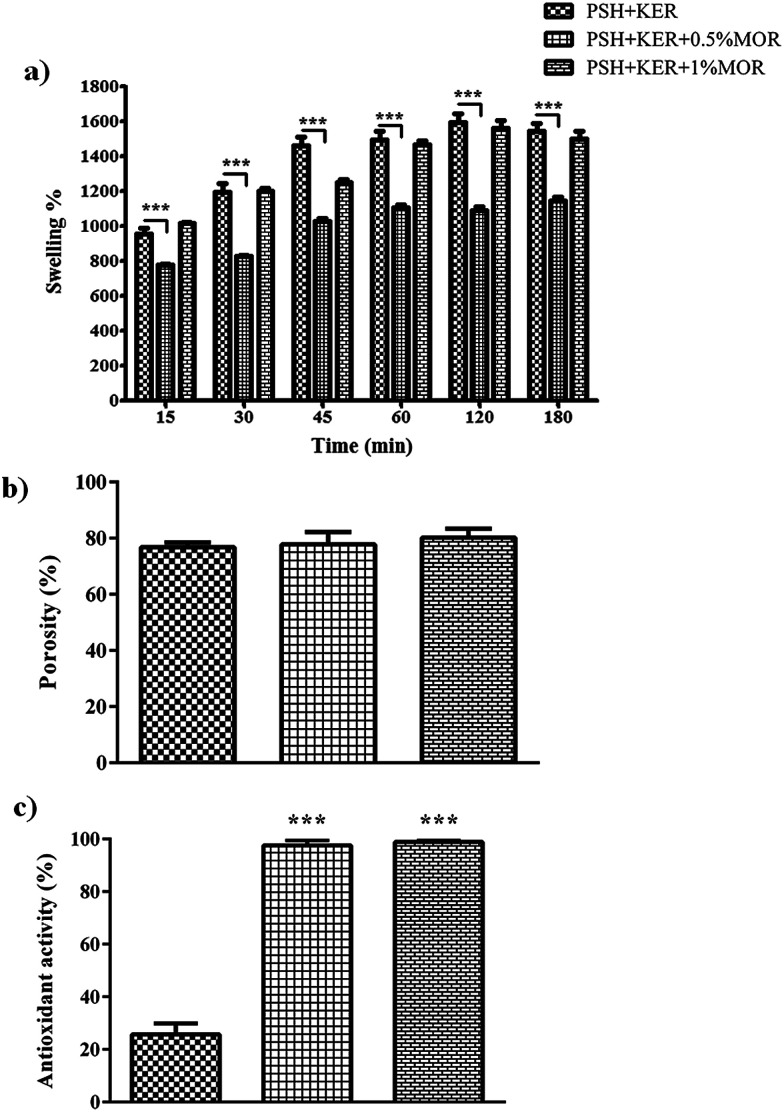
(a) Swelling, (b) porosity, (c) DPPH free radical scavenging assay. Values are stated as mean ± SD (*n* = 3) and the level of significance is denoted as ***p < 0.001 respectively, compared to the control.

Swelling increased up to 60 min in all the scaffolds. MOR incorporation caused a reduction in PBS uptake. PSH + KER + 0.5% MOR and PSH + KER + 1% MOR scaffolds exhibited 1100%, and 1400% swelling, respectively. The control scaffold, PSH + KER, displayed a slightly higher swelling of 1500% (Fig. 3a). Swelling capabilities of the scaffolds, as measured by a PBS absorption assay, is indicative of significant fluid uptake capabilities. These scaffolds may be suited for medium exuding wounds, with the capability to absorb discharges, and thereby prevent bacterial infections. These scaffolds can also facilitate moist wound healing, and thus accelerate the healing process. However, it was observed that the incorporation of morin reduces the swelling capabilities of the scaffolds. The porosity of the scaffolds measured by ethanol displacement was between 76–82% in all scaffolds (Fig. 3b).

### Antioxidant assay

DPPH free radical scavenging abilities of the MOR scaffolds were so high that all scaffolds that had MOR showed significantly higher antioxidant activity compared to the control PSH + KER scaffold. The near-absolute disappearance of the free radical was observed in the case of both the scaffolds with MOR (Fig. 3c). The experiment was reattempted with solutions of increased DPPH concentration, but the assay was limited by the tendency of DPPH to get auto-bleached at higher concentrations. The near-absolute bleaching of DPPH, effected by PSH + KER + 0.5% MOR and PSH + KER + 1% MOR is indicative of the strong antioxidant capabilities of the scaffolds. Antioxidant properties of the wound dressing material complement the healing capabilities by eliminating inflammatory stress imparted by the presence of free radicals. An early exit from inflammation is essential to the successful and timely healing of any chronic wound.

### Antibacterial assay

**Fig. 4:**
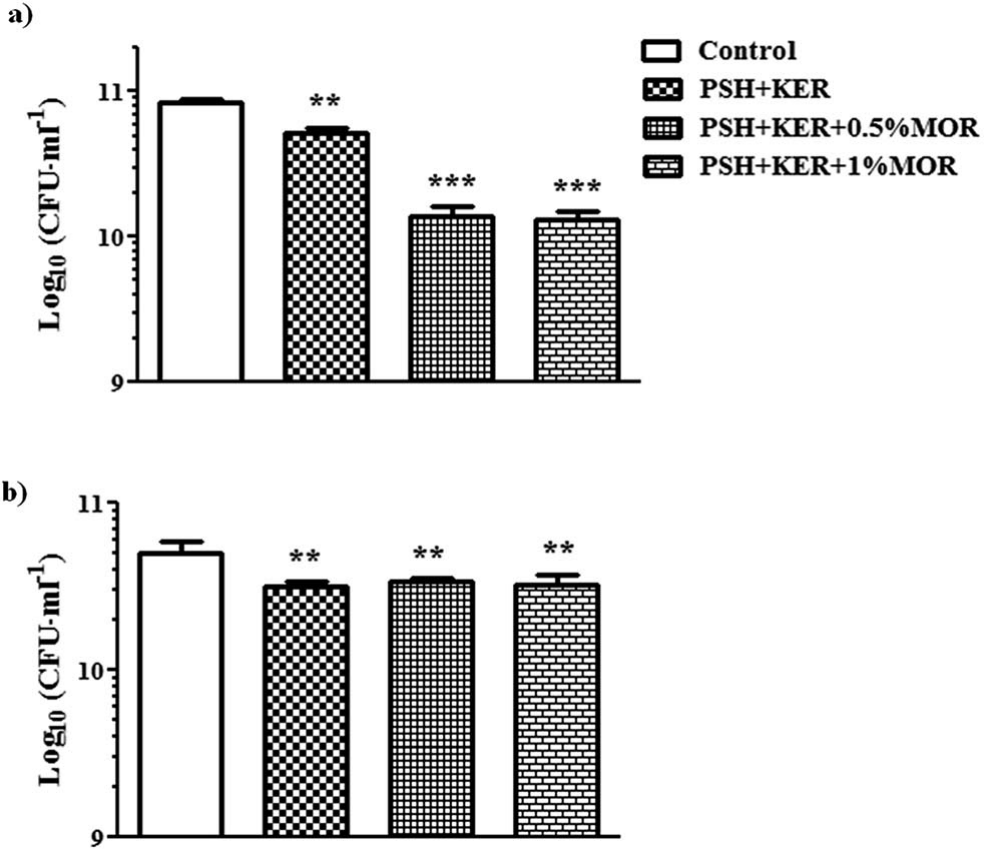
(a) Antibacterial activity of the fabricated scaffolds against *S. aureus*, (b) antibacterial activity of the fabricated scaffolds against *E. coli*. Values are stated as mean ± SD (*n* = 3) and the level of significance is denoted as **p < 0.01 and ***p < 0.001 respectively, compared to the control.

Antibacterial activity of the MOR scaffolds was assessed against both Gram-positive and Gram-negative bacterial representatives – *S. aureus* and *E. coli*, respectively. There is a significant bacteriostatic effect of the scaffold on *S. aureus* (Fig. 4a), and to a lesser extent on *E. coli*. However, the effect on *E. coli* is likely caused by the PSH + KER in the scaffolds rather than because of the MOR incorporated (Fig. 4b). Antibacterial assays on the MOR scaffolds show that they possess bacteriostatic capabilities. *S. aureus* growth appears to be retarded by the presence of MOR in the scaffolds. However, retardation of *E. coli* growth appears to be a consequence of the PSH + KER in the scaffold, rather than the MOR incorporated. Bacteriostatic properties of wound dressings can help in resisting possible infections at the site of the wound.

### Cell studies

**Fig. 5:**
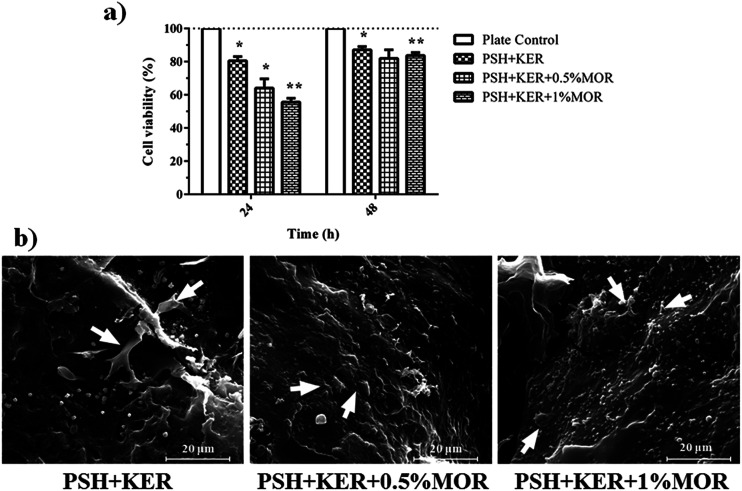
(a) Cell viability using Alamar blue assay, values are stated as mean ± SD (*n* = 3) and the level of significance is denoted as *p < 0.05 and **p < 0.01 respectively, compared to the control. (b) SEM images of cell attachment on the PSH + KER, PSH + KER + 0.5% MOR, and PSH + KER + 1% MOR scaffolds.

Cell viability was studied by measuring the reduction of resazurin by viable cells. In the first 24 h, there was a decreasing trend of the viability of the cells with increasing MOR concentration. The PSH + KER scaffold showed 80% cell viability in comparison to culture plate control. The PSH + KER + 0.5% MOR and PSH + KER + 1% MOR scaffolds displayed 65% and 57% viability, respectively. At 48 h, the cell viability increases to more than 82% in all the scaffolds, denoting a recovery of the cells. This could be explained by an acclimatization of the cells to the presence of the scaffolds and the burst release of MOR in the initial hours (Fig. 5a). SEM images of cells attached to the scaffolds were obtained to illustrate fibroblasts thriving on the scaffold surfaces by end of 48 h. The images show multiple fibroblast clusters with ECM constituents (Fig. 5b). Cell viability measured by the reduction of Alamar blue is indicative of an initial period of acclimatization of the fibroblast cells to the presence of MOR in the scaffolds. There is a significant drop in viability in the first 24 h of growth, which recovers by the end of 48 h. This indicates the recovery of cell growth, after a period of acclimatization to the initial burst of release of MOR from the scaffolds. SEM images of fibroblast attachment to the scaffolds confirm the inclination of the fibroblasts to attach on the scaffold surfaces.

### *In vivo* experiments

#### Wound contraction and period of epithelialization

Diabetic wounds pose a series of challenges in wound healing treatment. The underlying mechanism for impaired wounds in diabetes mellitus is still unclear. Clinically, an ideal wound dressing material is required with appropriate antioxidant, antimicrobial and anti-inflammatory properties to treat the diabetic wounds effectively. In order to achieve an ideal wound dressing material, morin was incorporated in a polysaccharide (psyllium husk) and protein (keratin) based scaffold. We have used streptozotocin induced diabetic rats for the wound healing experiment.

Wound healing is a complex biological process which occurs to restore the structural integrity of the damaged tissue.^46^ Inflammatory phase is characterized by the infiltration of polymorphonuclear neutrophils, macrophages, and lymphocytes at the wound site.^47^ In the present study, we employed morin as an anti-inflammatory agent to reduce the inflammation and subsequently improve the wound healing in diabetic rats.

**Fig. 6:**
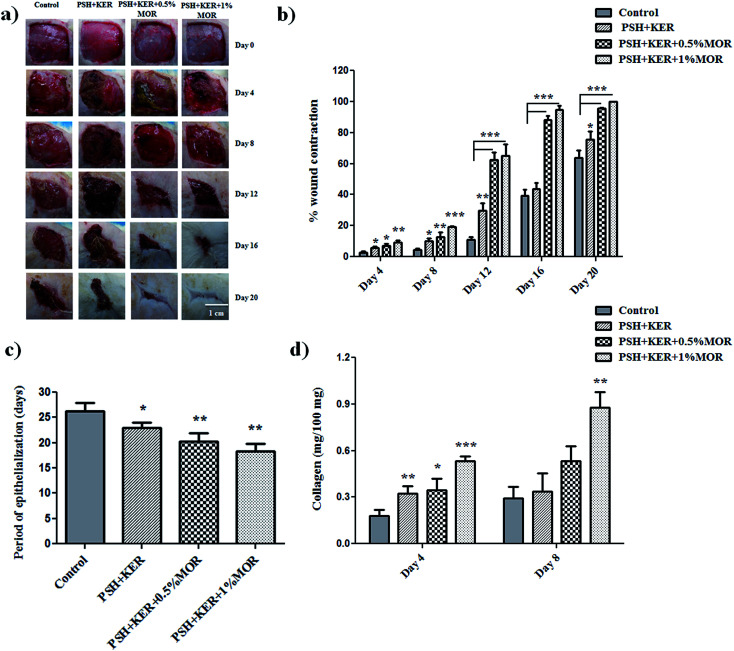
(a) Digital images of wound contraction, (b) percent wound contraction, (c) period of epithelialization and (d) collagen content. Values are stated as mean ± SD (*n* = 5) and the level of significance is denoted as *p < 0.05, **p < 0.01 and ***p < 0.001 respectively, compared to the control.

The rate of wound healing was regularly observed in the experimental rats from day 0 to complete healing to authenticate the wound healing property of untreated (control), PSH + KER, and PSH + KER + MOR scaffolds treated wounds. Wound size reduction was traced, captured on every 4-day interval and percentage wound contraction was calculated planimetrically (Fig. 6a and b). Diabetic control wound (group I) showed around 3% of wound contraction on day 4, 4% on day 8, 11% on day 12, 39% on day 16 and 64% on day 20. PSH + KER scaffold treated (group II) diabetic rats showed slightly increased wound contraction rate on days 4 (6%), 8 (10%), 12 (29%), 16 (44%) and 20 (76%) compared to control wounds. PSH + KER + 0.5% MOR scaffold treated diabetic rats exhibited a significantly higher rate of wound contraction compared to control and PSH + KER scaffold treated groups. PSH + KER + 0.5% MOR scaffold treatment (group III) showed 7% on day 4, 13% on day 8, 63% on day 12, 88% on day 16 and 95% on day 20. However, PSH + KER + 1% MOR treated wounds (group IV) showed faster wound contraction around 95% on day 16. PSH + KER + 1% MOR scaffold treated wounds showed 9% on day 4, 19% on day 8, 65% on day 12 and 95% on day 16. Hence, PSH + KER + 1% MOR scaffold dressing could be beneficial to treat the diabetic wounds. As the equilibrium between the collagen synthesis and degradation is maintained, we expect that faster contraction rate will not cause excessive scar formation.

Period of epithelialization was monitored and showed in Fig. 6c. Diabetic control wound showed around 26 days and PSH + KER scaffold dressing exhibited 23 days (*p* < 0.05) for complete wound closure. However, PSH + KER + 0.5% MOR, PSH + KER + 1% MOR scaffolds treatment displayed 20 and 18 days respectively (*p* < 0.001) for complete healing. Results revealed that morin incorporation significantly decreased the epithelialization time and accelerated the rate of wound contraction in diabetic rats. Epithelialization is an essential event required to reconstruct injured epithelial surface.^48^ Keratinocyte is a major cell type involved in the re-epithelialization process for the complete wound closure.^48^ Results of the present study reveal that the wound re-epithelialization was significantly faster in morin incorporated scaffolds treatment.

#### Collagen content

Wound granulation tissues collected from wound surface on days 4 and 8 were used to estimate hydroxyproline content. Hydroxyproline was used as a marker to quantify the amount of collagen present in granulation tissues (Fig. 6d). PSH + KER and PSH + KER + MOR scaffolds treated wound granulation tissues showed a high amount of collagen on days 4 and 8. Compared to control, the collagen content on PSH + KER scaffold treated wound tissues was significantly higher on day 4 (45% increase). However, on day 8, there was only a small increase in the collagen concentrations in the PSH + KER scaffold treated wounds, compared to control. In the case of PSH + KER + 0.5% MOR and PSH + KER + 1% MOR scaffold treatments, the collagen contents were much higher on both day 4 (increases of 48% and 67%, respectively) and day 8 (increases of 44% and 66%, respectively). Collagen is a major structural protein, its synthesis and deposition determine the rate of wound healing during wound repair and regeneration process.^49^ Collagen synthesis was significantly higher in morin loaded scaffolds (PSH + KER + MOR) treatment compared to PSH + KER scaffolds treated wounds and control wounds. Accelerated synthesis, deposition of collagen restores the structural and functional integrity of the injured tissue. Increased collagen deposition was observed in the PSH + KER + MOR scaffolds treatment.

#### Histopathology

**Fig. 7:**
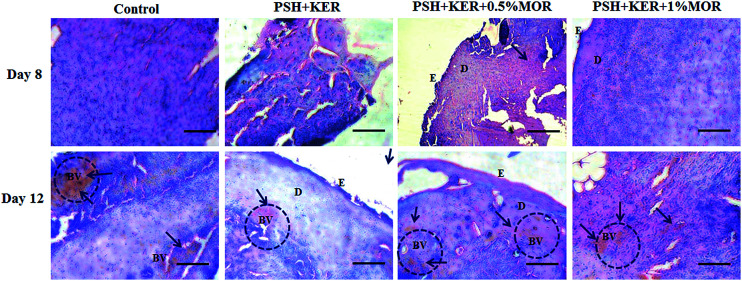
Histopathology studies of diabetic wounds. Hematoxylin and eosin (H&E) stained wound granulation tissues on days 8 and 12. Blue arrows denote the blood capillaries. E denotes the epidermis development, BV denotes the blood vessels, and D denotes the dermis layer. Scale bar 100 μm; magnification 20×.

Histopathology analyses helped us to understand the cellular activities during wound healing in experimental wounds. Wound granulation tissues collected on days 8 and 12 from control and treated wounds were used for the histopathological study. Granulation tissue sections were stained with hematoxylin and eosin (H&E), and Masson's trichrome stains. Cellular activities during wound healing cascades were microscopically observed using H&E staining. Synthesis, maturation, and deposition of collagen fibers were qualitatively assessed using Masson's trichrome staining. H&E stained tissue sections of days 8 and 12 were given in Fig. 7.

PSH + KER scaffold treated diabetic granulation tissues showed a few inflammatory cells on day 8. A thin epithelial layer and a few blood vessels were observed on day 12. PSH + KER + 0.5% MOR treated diabetic wounds displayed a few inflammatory cells, a thin epithelial layer and a large number of blood vessels on days 8 and 12. PSH + KER + 1% MOR showed a thin epithelial layer with a large number of infiltrated polymorphonuclear cells on day 8. Also, PSH + KER + 1% MOR treated tissues were observed with a large number of blood capillaries on day 12.

**Fig. 8:**
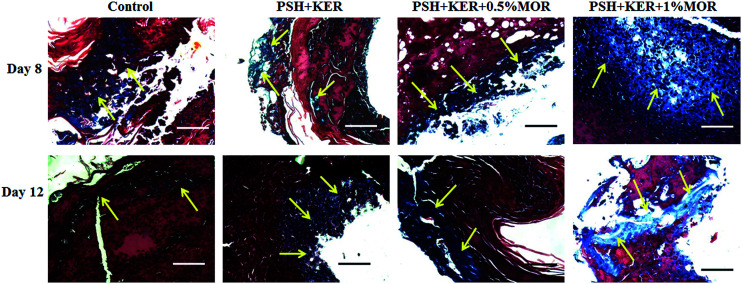
Collagen synthesis and deposition were assessed in wound granulation tissues on days 8 and 12 by Masson's trichrome staining. Yellow arrows represent the deposition of collagen fibers. Scale bar 100 μm; magnification 20×.

Masson's trichrome stained tissue sections showed the collagen synthesis and deposition in control and experimental groups on days 8 and day 12 (Fig. 8). Control tissue showed very less amount of collagen synthesis and deposition on days 8 and 12. Compared to control, PSH + KER scaffold treatment showed slightly increased collagen synthesis and deposition on days 8, and 12. However, morin (0.5% and 1%) loaded PSH + KER scaffolds treated tissues exhibited much higher collagen synthesis and deposition on days 8 and 12. Increased collagen content was also observed in collagen estimation on day 8 tissues (Fig. 6d). Overall, microscopical analyses showing that morin incorporated PSH + KER scaffolds improved the collagen synthesis, deposition, and blood vessel formation to accelerate the diabetic wound healing.

Wound repair process was monitored by the cellular activities involved in the wound healing cascades. This was assessed by the histopathological signs. H&E stained control and experimental wound granulation tissues were examined to understand the cellular activities, re-epithelialization, and collagen deposition. Control tissues show excess infiltration of polymorphonuclear neutrophils till day 12 responsible indicating the prolonged inflammatory phase in diabetic wounds. However, PSH + KER + MOR scaffolds treatment reduced the neutrophils infiltration on day 8 and promoted the wound healing. This is probably due to the presence of morin in the scaffolds. Morin provides ameliorative activity against reactive oxygen species (ROS) and intrinsic free radical scavenging activity. Also, morin suppresses the inflammatory cytokines leukotriene B4, nitric oxide, and interleukin-1β to reduce the severe inflammation.^50^ Overall, PSH + KER + MOR scaffolds treatment accelerated wound contraction, re-epithelialization, collagen synthesis and deposition significantly in diabetic rats, resulting in faster healing of diabetic wounds.

## Conclusions

We have fabricated a wound dressing material from a blend of psyllium seed husk polysaccharide and human hair-derived keratin and loaded it with morin. The hydrogel scaffolds were produced using freeze-drying, with STMP chemical crosslinking. Morin, a flavonol, shows exceptional antioxidant capabilities. PSH + KER, PSH + KER + MOR (0.5 and 1.0%) scaffolds showed good swelling, and porosity properties. In addition, PSH + KER + MOR (0.5 and 1.0%) scaffolds exhibited significant antioxidant and antibacterial properties compared to PSH + KER scaffolds. The cell viability and cell attachment studies showed that PSH + KER, PSH + KER + MOR (0.5 and 1.0%) scaffolds are biocompatible. *In vivo* diabetic wound healing study explored that PSH + KER + MOR 1.0% scaffold dressing could be beneficial for the faster re-epithelialization, collagen synthesis, and wound contraction in diabetic rats.

## Conflicts of interest

The authors have no conflicts of interest.

## Supplementary Material

RA-008-C7RA10334D-s001
