# Supplementary material for: Morin incorporated polysaccharide–protein (psyllium–keratin) hydrogel scaffolds accelerate diabetic wound healing in Wistar rats
Source: RSC Adv. 2018 Jan 9;8(5):2305–14. doi: 10.1039/c7ra10334d (PMC9077386; doi:10.1039/c7ra10334d)
Supplement: RA-008-C7RA10334D-s001 [file RA-008-C7RA10334D-s001.pdf]

Electronic Supplementary Material (ESI) for RSC Advances.
This journal is © The Royal Society of Chemistry 2018

Morin incorporated polysaccharide-protein (psyllium-keratin) hydrogel scaffolds accelerate diabetic wound healing in Wistar rats

Thangavel Ponrasu,[a] Praveen Krishna Veerasubramanian,[a] Ramya Kannan,[a,b] Selvakumar Gopika,[c] Lonchin Suguna,[c] and Vignesh Muthuvijayan[a*]

[a]Department of Biotechnology, Bhupat and Jyoti Mehta School of Biosciences, Indian Institute of Technology Madras, Chennai 600036, India.

[b]Department of Chemistry, Indian Institute of Technology Madras, Chennai 600036, India.

[c]Department of Biochemistry, CSIR-Central Leather Research Institute, Council of Scientific and Industrial Research, Adyar, Chennai 600020, India.

Corresponding Author: Email: vigneshm@iitm.ac.in; Phone: +91-44-2257 4123; Fax: +91-44-2257 4102.

Supplementary information

Mechanical strength

The compressive strength of the PSH+KER, PSH+KER+0.5%MOR and PSH+KER+1%MOR scaffolds were performed at room temperature on an E.Z test analyzer (SHIMADZU). Uniaxial compression testing was done for the scaffolds with a load cell of 500 N at a loading rate of 2mm/min. The compression moduli were calculated as elastic modulus using HOOKE's law model and the values were reported for the different concentration of the scaffolds (Table S1).[1]

Table S1. Mechanical strength

Sample	Elastic modulus (KPa)
PSH+KER	82.1
PSH+KER+0.5%MOR	50
PSH+KER+1%MOR	40.5

Loading efficiency

Morin loading efficiency in the scaffolds was performed by dissolving the scaffolds in 0.1 M NaOH under agitation for 1 h. The absorbance of the solution was measured at 387 nm. The concentration of MOR in the solution was calculated by consulting a morin standard curve that was prepared by measuring the absorbance (at λ=387 nm) of solutions of 0.025 – 0.25 mg/mL of MOR in PBS. Loading (in %) was calculated as the ratio of amount of morin measured in the scaffold to the amount of morin added to the scaffold (Figure S1).[2]

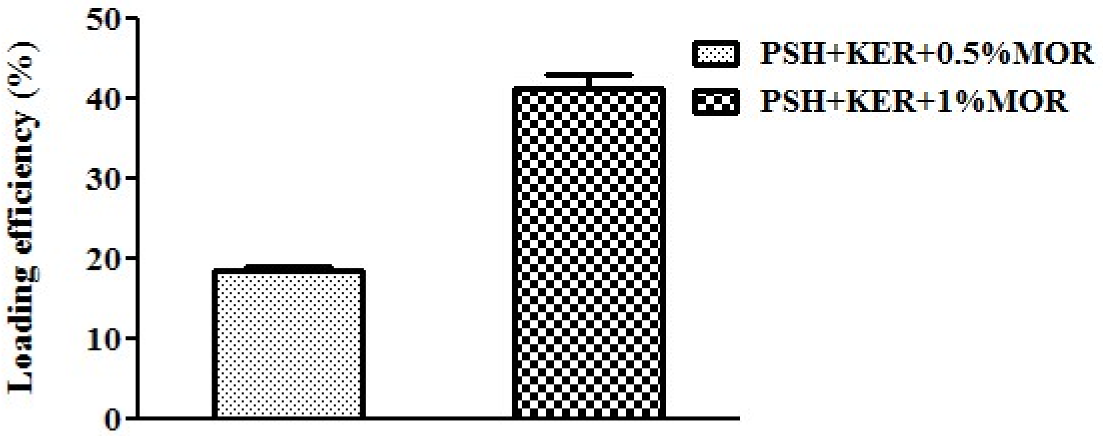

Figure S1. Loading efficiency

Release profile

Release of MOR from the scaffolds was measured by incubating known amounts (100 mg) of MOR scaffolds in 30 ml of PBS at 37°C, and withdrawing 1 ml of solution periodically. The withdrawn volume was replaced with fresh PBS. The concentration was calculated by measuring the absorbance of the solutions at 387 nm. Release (%) was calculated as the amount of MOR in solution to the amount of MOR loaded in the scaffold under testing (Figure S2).[3]

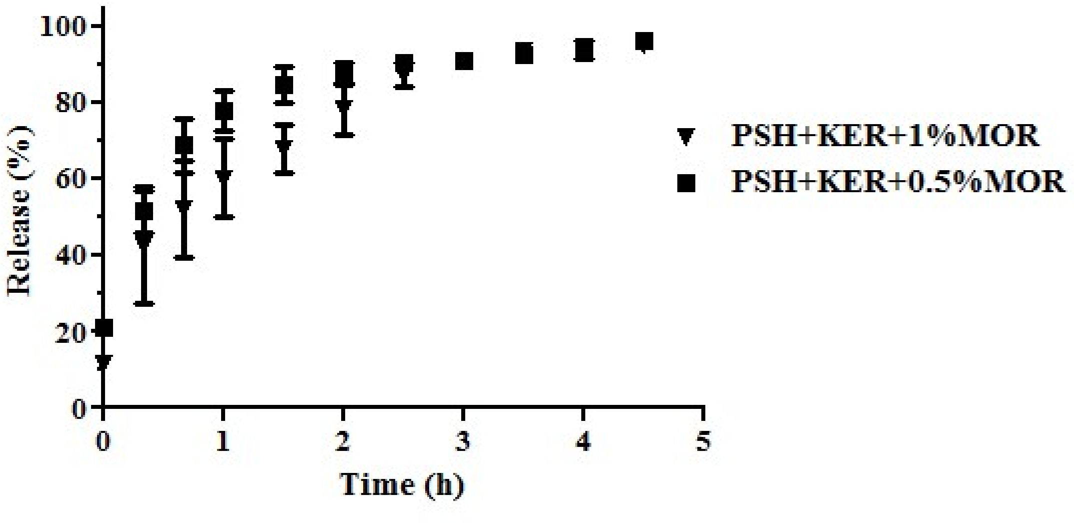

Figure S2. Release study

In vitro biodegradation

The *in vitro* biodegradation of the scaffolds was studied by placing the scaffolds in PBS solution (pH 7.4) containing 1 mg/mL lysozyme at 37°C. Briefly, scaffolds were equally weighed, soaked in PBS solution, pH 7.4 and incubated at 37°C for 60 days with shaking speed of 100 rpm. Scaffolds were removed at regular interval and rinsed thoroughly with deionized water to remove ions adsorbed on the surface and lyophilized. The dry weight of the each scaffold was weighed as W_t and initial weight as W_i. The biodegradation of scaffolds was calculated using the formula (Figure S3).[3]

$$\text{Biodegradation (\%)} = (W_i - W_t) / W_i \times 100$$

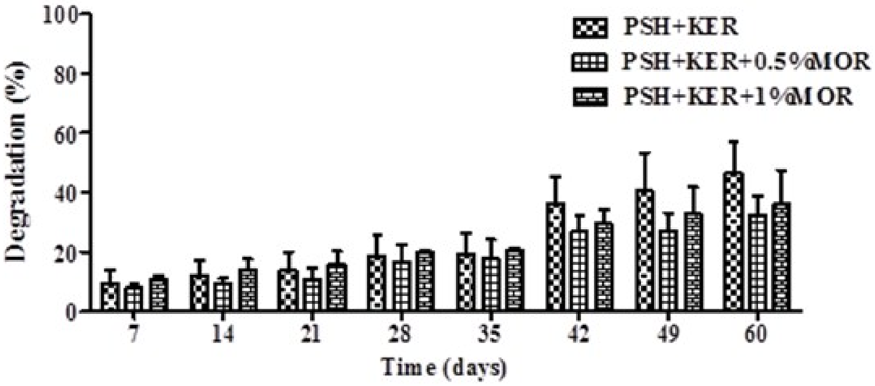

Figure S3. *In vitro* biodegradation

References

1. I. Ganesh, A. Venkata, Z. Yishan, L. Christopher and S. Susan, *Bioinspired, Biomimetic and Nanobiomaterials*, 2016, 5, 12-23.
2. L. H. Nguyen, M. Gao, J. Lin, W. Wu, J. Wang and S. Y. Chew, *Scientific Reports*, 2017, 7, 42212.
3. P. Thangavel, B. Ramachandran, S. Chakraborty, R. Kannan, S. Lonchin and V. Muthuvijayan, *Scientific Reports*, 2017, 7, 10701.
